# *Babylonia spirata* (Linnaeus, 1758) on biochemical and nutritional composition levels are altered by *Aeromonas hydrophila* infection

**DOI:** 10.1016/j.bbrep.2020.100746

**Published:** 2020-02-26

**Authors:** Gurusamy Chelladurai, Venkatasamy Uma

**Affiliations:** a PG & Research Department of Zoology, V.O.Chidambaram College, Tuticorin, TamilNadu, India; b Department of Botany, G.V.N College, Kovillpatti, Tamilnadu, India

**Keywords:** *Babylonia spirata*, Bacterial infection, Immune response, Biochemical composition, DNA assay

## Abstract

The present study comprises the biochemical and nutritional composition level of control and infected host of *B. spirata* with *A. hydrophila*. The healthy species were collected from the Therespuram coast, Southeast coast of India. After the acclimatization period, 15 snails were selected and infected with seven different bacterial pathogens by intramuscular injection. The snails which shows the maximum mortality rate after the bacterial infection was selected for the biochemical composition nutritional level. It was then analyzed and compared to the control group. Based on this result, the FTIR spectrum, DNA fragmentation, SDS PAGE Profile, amino acids (phenylalanine), fatty acids (linolenic acids), minerals (aluminum and copper) was recorded maximum in control and minimum in infected tissue of *B. spirata*. The result of the present study showed, presences of rich nutrition composition good protein profile in this species add more value of economic importance.

## Highlights

• This is new work; no work has been done in India.
• The *Aeronomons* sp has severely damage the snail population under laboratory condition.
• Studied nutritional composition between control and infected snail tissue.

## 1 Introduction

The gastropods have become adapted to almost every kind of existence on earth. Especially, spiral babylone (*Babylonia spirata),* a commercially important edible marine gastropod is observed to inhabit in the marine coastal waters in sandy benthic zones [1]. It's commonly known as whelks and easily caught and landed in shrimp trawlers of Kerala and Tamil Nadu. It is traditionally exploited for the shell-craft industries of Tamil Nadu. However, in early 90s the whelk fisheries have gained its significance, because of the demand in exporting its meat to certain Asian countries like, Japan and Singapore. The proximate composition has five basic constituents such as protein, carbohydrate, lipids, ash and moisture. Generally, nutrition refers to “nourishing or being nourished. Especially, it includes certain series of processes by which an organism takes in and assimilates for promoting growth and replacing worn out or injured tissue [2]. They are the important biochemical compounds necessary for all the biological activities of any living system. The cost of fish feed has been recognized as a major factor affecting the development and expansion of aquaculture enterprise in our country as well as other countries too [3]. The constituents of feed ingredients include fish meal, soybean meal, groundnut oil cake and wheat flour etc, which were also utilized for human consumption and also in the livestock industry. Therefore, it leads to the drastic decline in fish and livestock production (due to the high cost of feeding) with certain short fall in their protein intake in many developing countries [1]. Even though a large number of marine gastropods are suitable for human consumption, our knowledge of its nutritive value is fragmentary and a great deal of work is needed on the biochemical composition of marine gastropods which are fit for human consumption. In recent years, the application of vaccination and immunostimulants for disease management and prevention of diseases in shellfish aquaculture have been increasingly recognized as a promising new strategy. In general, immunostimulants comprise a group of biological and synthetic compounds that may enable to enhance the non-specific defence mechanisms in animals. Thereby, it imparts a generalized protection, particularly in the fishes that are raised in or released into environments where, the nature of pathogen is unknown. The immunization with specific vaccine may be futile at many instances [5,6]. Recently studied species are continuously exploited in traditional fishing area and this resulted in increased demand and higher price in current years. Form the aquaculture point of view, the Babylonia species needs study of biological attributes, production and market value. For a profitable aquaculture venture this may be considered a promising new candidate in land based aquaculture industry in India. The present study deals with the biochemical and nutritional levels among the control and infected tissue of *B. spirata.*

## 2 Materials and methods

### 2.1 Collection of species

The samples of *B. spirata* with their initial weight ranged from 30.18 g to 31.11 g and initial length ranged from 3.14 cm to 3.85 cm were collected from the Therespuram coastal area (80^0^ 48′N; 78^0^ 94′ E), Tuticorin, Southeast coast of India.

### 2.2 Experimental setup

Collected samples were made to acclimatize for 7 days in by using aerated plastic holding tanks (1.5 m × 2 m × 0.5 m L: W: H) in the Marine Gastropod Hatchery Research Laboratory, Kamaraj College, Tuticorin, Tamil Nadu, India. Then they were randomly distributed into triplicate FRP (Fibreglass Reinforced Plastics) tank containing 500 L at about 40 snail/tank and three experimental groups were maintained. The tanks were regularly cleaned, disinfected and allowed to dry for 24 h after which they were filled with dechlorinated ambient seawater up to 2/3 size of the tanks. The bottom of the rearing tanks was covered with 3 cm layer of coarse sand (500–1000 μm mean grain size) as substrate. During the study period, the snails were fed with natural live clam meat at once a day. The seawater quality parameter was analyzed every day for its purity. It includes parameters like, temperature (^0^C), salinity (ppt), pH and dissolved oxygen (mg/L) were examined by using SYSTRONICS water analyzer 371. The total experiment was conducted for 7 days.

### 2.3 Microbial culture

The bacterial strains namely, *Aeromonas hydrophila* (IDH 1585), *Bacillus subtilis* (MTCC 441), *Vibrio harveyi* (MTCC 3438), *Vibrio parahaemolyticus* (J13300), *Escherichia coli* (H10407), *Staphylococcus aureus* (MTCC 1789) and *Vibrio cholera* (IDH5439) were selected on their pathogenicity and procured the bacterial strains were clinical isolates obtained from the Microbial Type Culture Collection (MTCC), Institute of Microbial Technology, Chandigarh, India.

### 2.4 Preparation of inoculums

Nutrient broth (Himedia M002500G) was prepared and 5 ml of this broth was poured in each of the 10 ml test tubes sterilized in an autoclave at 121 °C for 15 min. After which, these test tubes containing the sterile broth were inoculated with the six species of bacterial strains and incubated at 37 °C for 24 h. Triplicates were maintained.

### 2.5 Bacterial challenges

The bacterial challenge method was followed [7] after acclimatization, a random sample (N = 15) of *B. spirata* were taken from each treatment and transferred into 50 L plastic tanks. The water supply passed through dechlorinated seawater at 0.51/min maintained. The snails were challenged with seven bacterial pathogens at the load of 1 × 10^6^ CFU/ml and injected (0.5 ml) in their muscular foot. The control group was injected with 0.5 ml of physiological saline solution. All groups were kept under the observation for 7 days. The clinical symptomatic signs and daily mortality rate were recorded. The maximum mortality rate shown groups were selected for biochemical and nutritional levels analysis. It was then compared with their control groups.

The eight experimental groups were follows.

C - Control (uninfected)
T_1-_*Bacillus subtilis*
T_2-_*Staphylococcus aureus*
T_3-_*Escherichia coli*
T_4-_*Vibrio harveyi*
T_5_ - *Vibrio cholera*
T_6_ - *Vibrio paraheamolyticus*
T_7-_*Aeromonas hydrophila*

### 2.6 Determination of protein molecular weight

The molecular gel separation of (SDS-PAGE) the wet tissue of wild, control and post challenge tissue of *B. spirata* was carried out by the method [8]. The glass plates were assembled and 20 ml of 15% resolved gel was prepared and poured immediately to the notch plate. It was completed, overlay was poured off and the top layer was washed with distilled water. Then 8 ml of stock gel was over laid. Approximately volume of 1% SDS gel loading buffer and samples was taken and heated at 100 °C for 3 min. Then it was fixed in electrophoresis apparatus and 15 μL of sample and marker (14.3–97.4 KDa) was loaded respectively in the well. The gel was run and stained with observed under gel.

### 2.7 DNA fragmentation analysis

The tissue of wild, control and infected *B. spirata* were collected for their DNA extraction. Primarily, tissue samples were homogenized with lysis buffer and to that 2 ml of phenol (neutralized with TE buffer, pH 7.5) were added. Then, it was followed by the addition of 1 ml of chloroform and Isoamyl alcohol in the ratio of 24:1. Then ice cold ethanol and 10% sodium acetate in ratio of 2:5 was mixed. The precipitate form was stored at −20 °C over night. After centrifugation at 13,000 rpm for 10 min the pellets were air dried and were suspended with 50 μL of TF buffer containing 0.5 μL of ethidium bromide. After electrophoresis, the gel was photographed and UV light. The DNA fragmentation analysis was followed by the method [9].

### 2.8 Estimation of amino acids

The collected tissues were dried at 60 °C for 24 h in an oven and they were packed in airtight polyethylene covers and kept in desiccators. The oven dried samples were finely grounded before estimating amino acid profile. Amino acids were estimated in HPLC-Lachrome merck in SPD-10 A VP Detector. The amino acid composition analysis was carried out [10].

### 2.9 Estimation of fatty acids

The samples were oven dried at 70 °C for 24 h until no more weight reduction was observed. After that, it was grounded finely with pestle and mortar. To the 100 mg–200 mg of finely ground tissue samples, 2 ml of chloroform and methanol (1:1 ratio) was added and kept aside for 30 s. Then the residual matter was removed through filtration with the whatman no:1 filter paper (125 mm). After that, it was subjected to washing with 1 ml of chloroform and methanol (2:1 ratio) for removing the inorganic substance. Next, the extracts were infected with chloroform: methanol: water (8:4:3) where, residual phase were evaporated to dryness. Then the dried matter was sealed in a test tube with 3% methanolic HCl and stored at 80 °C for 18 h. To this 2 ml of hexane was added for extraction of the fatty acid ethyl esters from the methanol by hexane. 1 ml of the supernatant containing hexane phase was collected in a microvel. After which, the residual fraction was dissolved in the ratio of 10:1 with ethyl acetate and 1:1 aliquot of which was injected into a gas chromatography (Agilent 6890,1997) equipped with flame identification detector and column HP ULTRA -2 (25 m, 0.2 mm 1D). The fatty acid composition analysis was done by the following method [11].

### 2.10 Estimation of minerals

The concentration of the metals like calcium, sodium, magnesium, potassium, phosphorous, barium, iron, zinc, cobalt, chromium, copper, manganese, gallium, lithium, nickel, selenium, aluminium and boron were estimated from 1 g of mechanically grounded and oven dried at 175 °C. Then it was digested with sulphuric acid and 40% nitric acid and were allowed to stand overnight at room temperature, before being analyzed for specific metals, using Atomic Absorption Spectrophotometer (AAS) Shimadzu –AA-65015. The total mineral contents were estimated by the following the method [12].

### 2.11 Fourier transform infrared spectroscopy (FTIR) spectral analysis

The lyophilized (powder) samples of *B. spirata* (10 mg) were mixed with 100 mg of dried potassium bromide (Kbr) and compressed to prepare appropriate discs. The discs were then read spectrophotometrically (Bio-Rad FTIR-40 Model; USA) and the frequencies of different components present in the samples were analyzed.

### 2.12 Statistical analysis

All experimental data obtained were analyzed using one-way analysis of variance (ANOVA) followed by Duncan's multiple range test p < 0.05 was considered for describing the significant level (SPSS Version 20).

## 3 Result

### 3.1 Bacterial challenge test

After 7 days of intramuscular injection of seven pathogen significantly p < 0.05 different in survival rates were observed among the control and treatments (Table 1). The maximum survival rate was observed in T_1_ and T_2_ of *B. subtilis* (70%) and *S. aureus* (70%) and minimum survival rate was observed in T_7_ of *A. hydrophila* (70%). In further studies the highest mortality rate of *A. hydrophila* and control (without injected) were analyzed for nutritional status and immunomodulation activity**.**

**Table 1:** Survival rate of *B. spirata* infected with different pathogens.

S.No	Treatments	Test Bacterial Pathogens	Route of Injection	Mortality (%)	Survival (%)
1	Control	PBS	I/M	0	100
2	T_1_	*Bacillus subtilis*	I/M	30	70
3	T_2_	*Staphylococcus aureus*	I/M	30	70
4	T_3_	*Escherichia coli*	I/M	40	60
5	T_4_	*Vibrio harveyi*	I/M	40	60
6	T_5_	*V. cholera*	I/M	50	50
7	T_6_	*V. parahemolyticus*	I/M	50	50
8	T_7_	*Aeromonas hydrophila*	I/M	70	30

I/M = Intra Muscular.

### 3.2 Water quality parameters

The average water quality parameters observed after infected with *A. hydrophila* showed in Table 2. The temperature ranged between 26 ± 0.35 °C and 27.48 ± 0.47 °C, the salinity from 32.21 ± 0.21 ppt to 33.78 ± 0.17 ppt, the pH from 7.71 ± 0.28 to 8.03 ± 0.29 and the dissolved oxygen from 5.34 ± 0.33 mg ^−1^ to 6 ± 0.25 mg^−1^.

**Table 2:** Assessment of water quality parameters observed during infection period of *B. spirata* (Contd.).

Day	Treatments with pathogens	Temperature (^0^C)	Salinity(ppt)	pH	DO mg^−1^
1	Control	26.51 ± 0.52^a^	33.01 ± 0.21^ba^	7.82 ± 0.35^cd^	5.34 ± 0.33^d^
1	T_1_ (*B. subtilis)*	26.53 ± 0.38^a^	33.21 ± 0.29^b^	7.88 ± 0.31^ca^	5.38 ± 0.38^da^
1	T_2_ (*S. aureus)*	27.03 ± 0.35^ab^	33.51 ± 0.35^ba^	7.79 ± 0.34^c^	5.54 ± 0.29^da^
1	T_3_ (*E. coli*)	27.15 ± 0.37^a^	33.14 ± 0.41^ba^	7.95 ± 0.32^c^	5.63 ± 0.38^da^
1	T_4_ (*V. harveyi*)	26.81 ± 0.42^a^	33.69 ± 0.53^ba^	7.81 ± 0.28^c^	5.71 ± 0.31^d^
1	T_5_ (*V. cholera*)	26.35 ± 0.41^a^	32.98 ± 0.36^ba^	7.94 ± 0.24^c^	5.74 ± 0.28^da^
1	T_6_(*V.parahemolyticus)*	26.33 ± 0.38^a^	33.15 ± 0.29^ba^	7.81 ± 0.25^c^	5.63 ± 0.25^da^
1	T_7_ (*A. hydrophila)*	26.38 ± 0.37^a^	33.81 ± 0.25^bc^	7.99 ± 0.28^c^	5.56 ± 0.38^dc^
2	Control	26.31 ± 0.51^a^	32.21 ± 0.21^bc^	8.03 ± 0.29^ca^	5.81 ± 0.35^d^
2	T_1_ (*B. subtilis)*	26.11 ± 0.38^a^	33.15 ± 0.24^bc^	7.79 ± 0.30^c^	5.85 ± 0.28^dc^
2	T_2_ (*S. aureus)*	26.08 ± 0.41^ab^	33.14 ± 0.20^b^	7.81 ± 0.31^cb^	5.79 ± 0.27^da^
2	T_3_ (*E. coli*)	26.51 ± 0.47^a^	33.18 ± 0.31^ba^	7.80 ± 0.34^c^	5.81 ± 0.31^da^
2	T_4_ (*V. harveyi*)	26.53 ± 0.43^a^	33.41 ± 0.35^ba^	7.94 ± 0.32^c^	5.31 ± 0.24^da^
2	T_5_ (*V. cholera*)	26.38 ± 0.53^a^	33.38 ± 0.28^ba^	8.03 ± 0.31^c^	5.48 ± 0.30^da^
2	T_6_(*V.parahemolyticus)*	26.53 ± 0.71^a^	33.29 ± 0.33^ba^	7.95 ± 0.29^c^	5.35 ± 0.29^da^
2	T_7_ (*A. hydrophila)*	26.84 ± 0.63^a^	33.41 ± 0.27^ba^	7.63 ± 0.26^c^	5.48 ± 0.61^da^
3	Control	27.71 ± 0.52^ac^	33.15 ± 0.25^b^	8.01 ± 0.24^c^	5.71 ± 0.25^dc^
3	T_1_ (*B. subtilis)*	27.33 ± 0.27^a^	33.18 ± 0.19^bc^	7.81 ± 0.28^c^	5.79 ± 0.24^dc^
3	T_2_ (*S. aureus)*	27.14 ± 0.38^a^	33.14 ± 0.21^ba^	7.83 ± 0.25^c^	5.81 ± 0.21^da^
3	T_3_ (*E. coli*)	27.15 ± 0.52^a^	33.18 ± 0.34^bc^	7.81 ± 0.28^ca^	5.66 ± 0.31^da^
3	T_4_ (*V. harveyi*)	27.08 ± 0.38^a^	33.15 ± 0.25^bc^	7.94 ± 0.24^c^	5.61 ± 0.34^da^
3	T_5_ (*V. cholera*)	26.94 ± 0.57^ab^	33.78 ± 0.17^b^	8.03 ± 0.28^ca^	5.73 ± 0.35^d^
3	T_6_(*V.parahemolyticus)*	26.89 ± 0.48^ab^	33.17 ± 0.20^bc^	7.98 ± 0.29^ca^	5.72 ± 0.36^da^
3	T_7_ (*A. hydrophila)*	26.51 ± 0.38^ac^	33.00 ± 0.21^bc^	7.83 ± 0.25^c^	5.69 ± 0.33^da^
4	Control	26.66 ± 0.57^a^	32.89 ± 0.21^ba^	7.79 ± 0.41^c^	5.71 ± 0.27^da^
4	T_1_ (*B. subtilis)*	26.78 ± 0.82^ab^	32.89 ± 0.18^b^	7.81 ± 0.38^cb^	5.73 ± 0.29^db^
4	T_2_ (*S. aureus)*	27.01 ± 0.23^a^	33.04 ± 0.21^ba^	7.83 ± 0.40^c^	5.79 ± 0.18^da^
4	T_3_ (*E. coli*)	26.51 ± 0.55^a^	33.13 ± 0.41^ba^	7.81 ± 0.41^c^	5.81 ± 0.21^da^
4	T_4_ (*V. harveyi*)	26.80 ± 0.81^ab^	33.18 ± 0.35^b^	7.83 ± 0.35^cb^	5.89 ± 0.14^db^
4	T_5_ (*V. cholera*)	26.05 ± 0.32^a^	33.00 ± 0.38^bc^	7.91 ± 0.35^c^	5.68 ± 0.19^dc^
4	T_6_(*V.parahemolyticus)*	27.03 ± 0.26^ab^	33.18 ± 0.31^b^	7.82 ± 0.31^c^	5.94 ± 0.24^db^
4	T_7_ (*A. hydrophila)*	26.33 ± 0.31^ab^	33.19 ± 0.28^b^	7.85 ± 0.30^cb^	5.91 ± 0.23^d^
5	Control	27.07 ± 0.25^ac^	33.20 ± 0.31^b^	7.81 ± 0.23^c^	5.81 ± 0.27^dc^
5	T_1_ (*B. subtilis)*	27.32 ± 0.27^ab^	33.19 ± 0.28^bc^	7.85 ± 0.25^c^	6.00 ± 0.25^db^
5	T_2_ (*S. aureus)*	27.05 ± 0.87^ac^	33.12 ± 0.32^b^	7.85 ± 0.31^cb^	5.89 ± 0.31^d^
5	T_3_ (*E. coli*)	27.03 ± 0.37^a^	33.18 ± 0.30^b^	7.83 ± 0.24^c^	5.88 ± 0.25^d^
5	T_4_ (*V. harveyi*)	27.32 ± 0.81^a^	33.28 ± 0.31^b^	7.89 ± 0.28^c^	5.89 ± 0.19^d^
5	T_5_ (*V. cholera*)	27.11 ± 0.53^a^	33.04 ± 0.21^b^	7.85 ± 0.24^c^	5.80 ± 0.21^d^
5	T_6_(*V.parahemolyticus)*	27.35 ± 0.53^a^	33.21 ± 0.18^b^	7.71 ± 0.28^c^	5.79 ± 0.37^d^
5	T_7_ (*A. hydrophila)*	27.48 ± 0.47^a^	33.28 ± 0.32^b^	7.91 ± 0.23^c^	5.80 ± 0.33^d^
6	Control	26.50 ± 0.28^a^	33.12 ± 0.14^b^	7.89 ± 0.31^c^	5.91 ± 0.21^d^
6	T_1_(*B. subtilis)*	26.06 ± 0.37^a^	33.24 ± 0.18^b^	7.85 ± 0.32^c^	5.90 ± 0.24^d^
6	T_2_(*S. aureus)*	26.36 ± 0.22^a^	33.19 ± 0.21^ba^	7.91 ± 0.38^c^	5.89 ± 0.23^da^
6	T_3_ (*E. coli*)	26.35 ± 0.28^a^	33.15 ± 0.28^b^	7.96 ± 0.41^cb^	5.93 ± 0.28^d^
6	T_4_ (*V. harveyi*)	26.66 ± 0.31^ab^	33.13 ± 0.19^bc^	7.91 ± 0.39^c^	5.91 ± 0.31^da^
6	T_5_ (*V. cholera*)	26.28 ± 0.47^ab^	33.10 ± 0.25^ba^	7.89 ± 0.25^ca^	5.90 ± 0.25^d^
6	T_6_(*V.parahemolyticus)*	26.35 ± 0.21^a^	33.18 ± 0.21^ba^	7.84 ± 0.21^ca^	5.94 ± 0.20^d^
6	T_7_ (*A. hydrophila)*	26.00 ± 0.35^ab^	33.17 ± 0.19^b^	7.91 ± 0.24^c^	5.90 ± 0.19^db^
7	Control	27.31 ± 0.35^ac^	33.18 ± 0.15^bc^	7.81 ± 0.29^c^	5.90 ± 0.38^d^
7	T_1_ (*B. subtilis)*	27.08 ± 0.29^a^	33.27 ± 0.20^bc^	7.89 ± 0.25^c^	5.89 ± 0.31^dc^
7	T_2_ (*S. aureus)*	27.32 ± 0.38^ab^	33.21 ± 0.21^ba^	7.69 ± 0.24^ca^	5.91 ± 0.28^d^
7	T_3_ (*E. coli*)	27.14 ± 0.31^a^	33.19 ± 0.19^b^	7.83 ± 0.31^ca^	5.89 ± 0.25^d^
7	T_4_ (*V. harveyi*)	26.99 ± 0.43^a^	33.13 ± 0.20^ba^	7.94 ± 0.29^c^	5.94 ± 0.25^d^
7	T_5_ (*V. cholera*)	26.79 ± 0.29^a^	33.15 ± 0.26^ba^	7.89 ± 0.32^c^	5.91 ± 0.31^da^
7	T_6_(*V.parahemolyticus)*	27.33 ± 0.18^a^	33.19 ± 0.27^b^	7.83 ± 0.31^cb^	5.90 ± 0.24^d^
7	T_7_ (*A. hydrophila)*	27.21 ± 0.12^a^	33.20 ± 0.31^ba^	7.81 ± 0.25^c^	5.92 ± 0.35^da^

^abcd^ (Mean ± SD) the same letter in the same row is not significantly different at P < 0.05.

### 3.3 Molecular weight determination through SDS-PAGE analysis

**Fig. 1:**
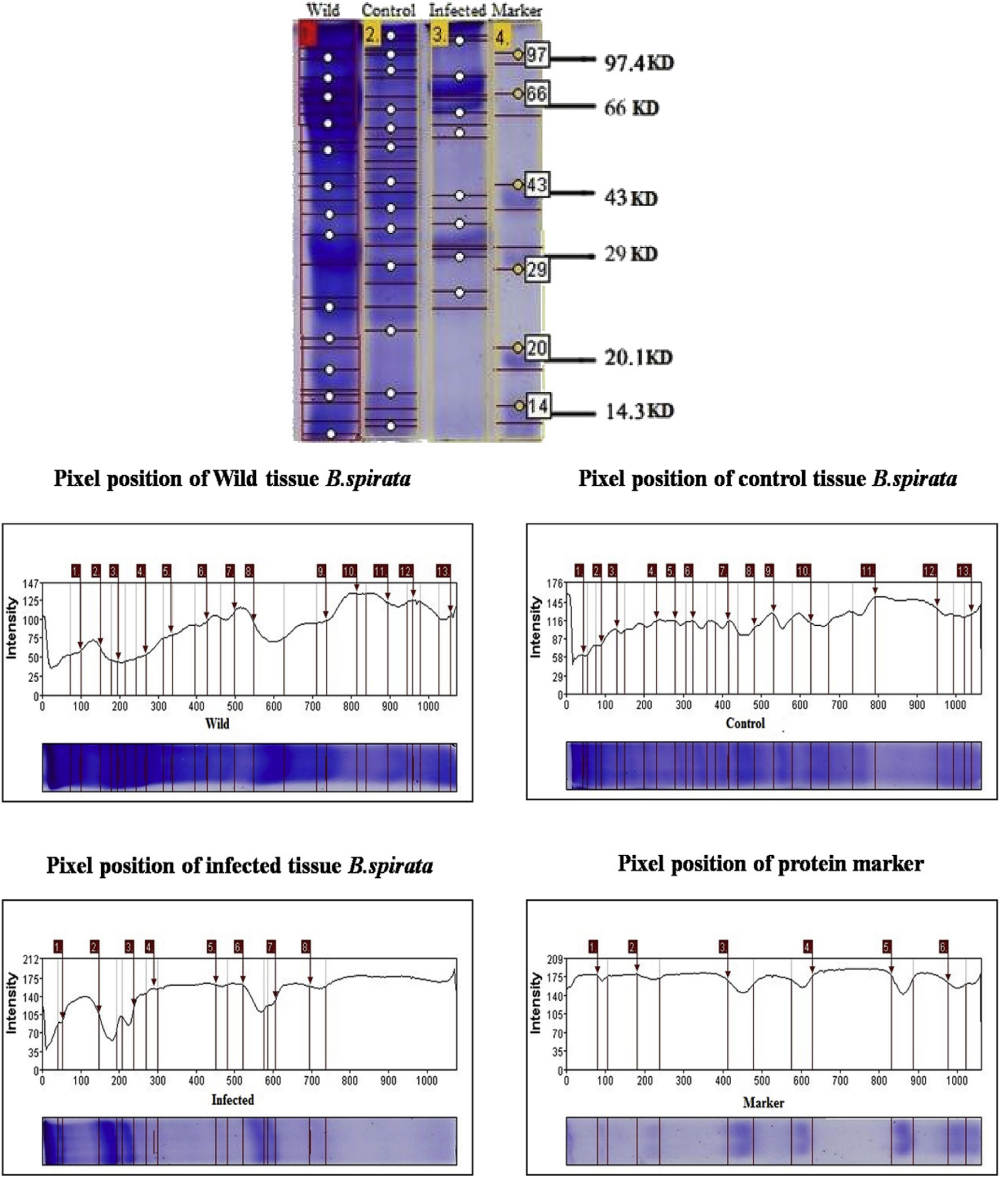
SDS –PAGE profile of wild, control and infected with *A. hydrophila* of *B. spirata*.

The SDS-PAGE gel profile and the pixel position of the bands obtained from the wild, control and infected protein marker from *B. spirata* are shown in Fig. 1. The gel obtained through SDS-PAGE showed wild tissue protein marker recorded 13 bands with total molecular weight ranged from 89 to 16 KDa. The control tissue protein marker recorded 13 bands with total molecular weight of 104 to 16 KDa. The infected tissue protein marker recorded only 8 bands with total molecular weight ranged from 102 to 21 KDa. The standard protein marker recorded 6 bands with the molecular weight of 97, 66, 43, 29, 20 and 14 KDa.

### 3.4 DNA damage analysis

**Fig. 2:**
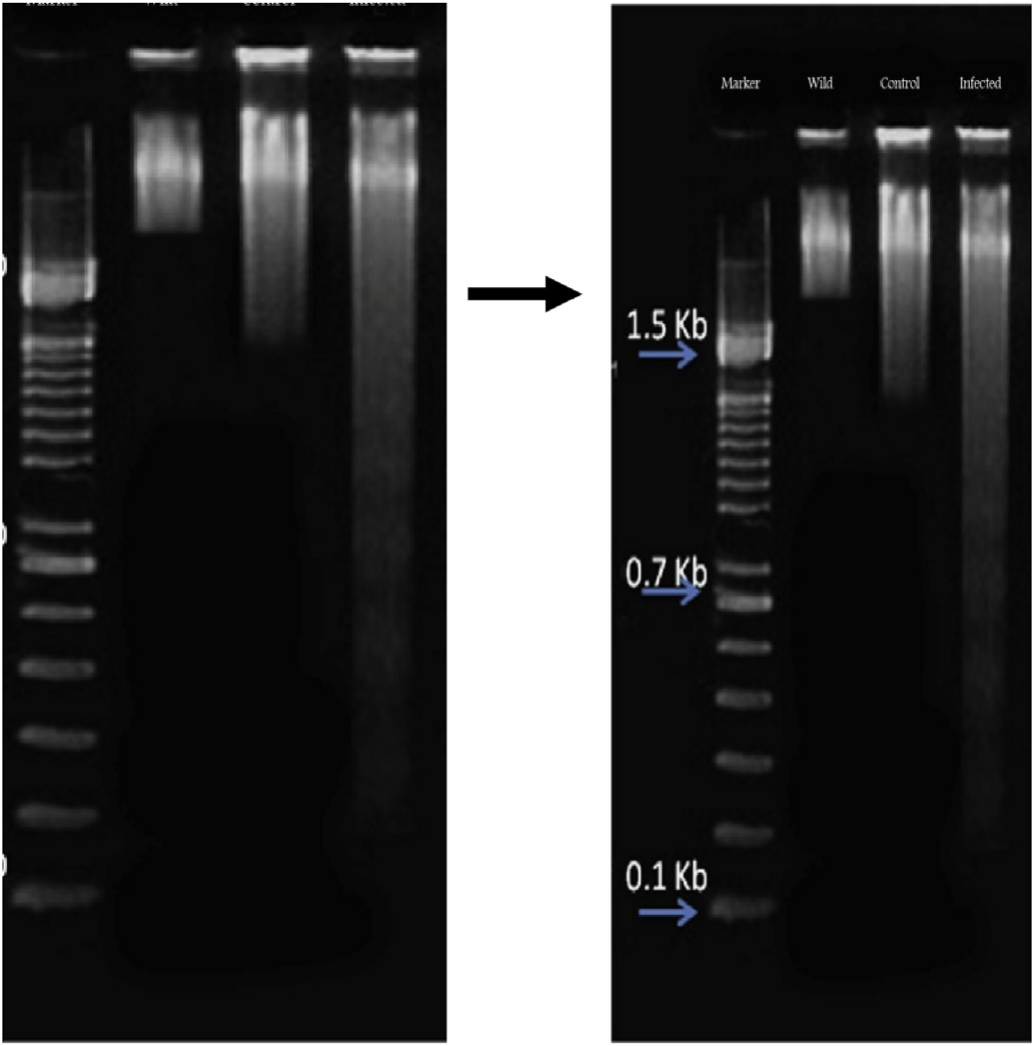
DNA fragmentation assay of wild, control and infected with *A. hydrophila of B. spirata*.

The DNA fragmentation is an obvious characteristic in cells entering apoptosis, the wild, control and infected bands were showed in Fig. 2. The wild and control tissue DNA samples showed very cleared thin bands with smear and DNA fragmentation was not observed. The infected tissue DNA samples showed the low bands were observed.

### 3.5 Fourier transform infrared spectroscopy (FTIR) spectral analysis

The FTIR spectra of control and post challenge tissue of *B. spirata* are presented in Table 3. In details spectral analysis were performed in the 400-4000 cm^−1^ region and the intensity of infected tissue increased than the control due to changes in the biochemical properties. The FTIR of control tissue was showed 19 major peaks lying between 3315.41 cm^−1^ and 603.68 cm^−1^, in which 1456.16 cm^−1^ indicated CH_2_ bend, 1400.22 cm^−1^ O–H bend, 1193.85 cm^−1^ C–C(O)–C stretch, 116.71 C–C-stretch, 750.26 cm^−1^ and 603.68 cm^−1^ and corresponded to CH bend, acetylenic C–H bend (Fig. 3a). Whereas the FTIR spectrum of infected tissue of *B. spirata* recorded totally, 15 major peaks lying between 3315.41 cm^−1^and 601.68 cm^−1^, in which 3315.41 cm^−1^indicated the O–H stretch, 1070.24 cm^−1^ indicated C=C stretch, 1403.22 cm^−1^ O–H bend, 114.78 cm^−1^ indicated C–C stretch, 748.33 cm^−1^ and corresponded to acetylenic C–H bend 1670.24 cm^−1^ corresponded to O–H stretching, 1456.16 cm^−1^indicated C=C stretch (Fig. 3b).

**Table 3:** FT-IR spectra: Vibration assignment of control and infected with *A. hydrophila* of *B. spirata*.

S. No.	Wave number cm^−1^		Appearance range	Bond	Assessment of functional groups
	Control tissue	Infected tissue			
1	3315.41	3315.41	Medium	O–H Stretch	Alcohol
2	1670.24	1670.24	Medium	C=C Stretch (isolated)	Alkene
3	1456.16	–	Weak to strong	CH2 bend	Alkane
4	1400.22	1403.22	Medium to strong	O–H bend	Carboxylic acid
5	1193.85	–	Medium	C–C(O)–C Stretch	Ester
6	1116.71	1114.78	Medium	C–C Stretch	Ketone
7	750.26	748.33	Strong	CH bend (ortho)	Aromatic
8	651.89	–	Weak to medium	Acetylenic C–H bend	Alkyne
9	603.68	601.68	Medium to strong	Acetylenic C–H bend	Alkyne

**Fig. 3a:**
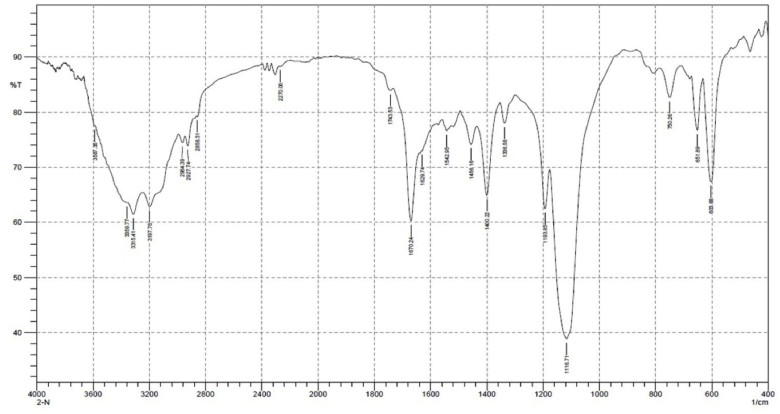
FT-IR spectrum of control tissue of *B. spirata*.

**Fig. 3b:**
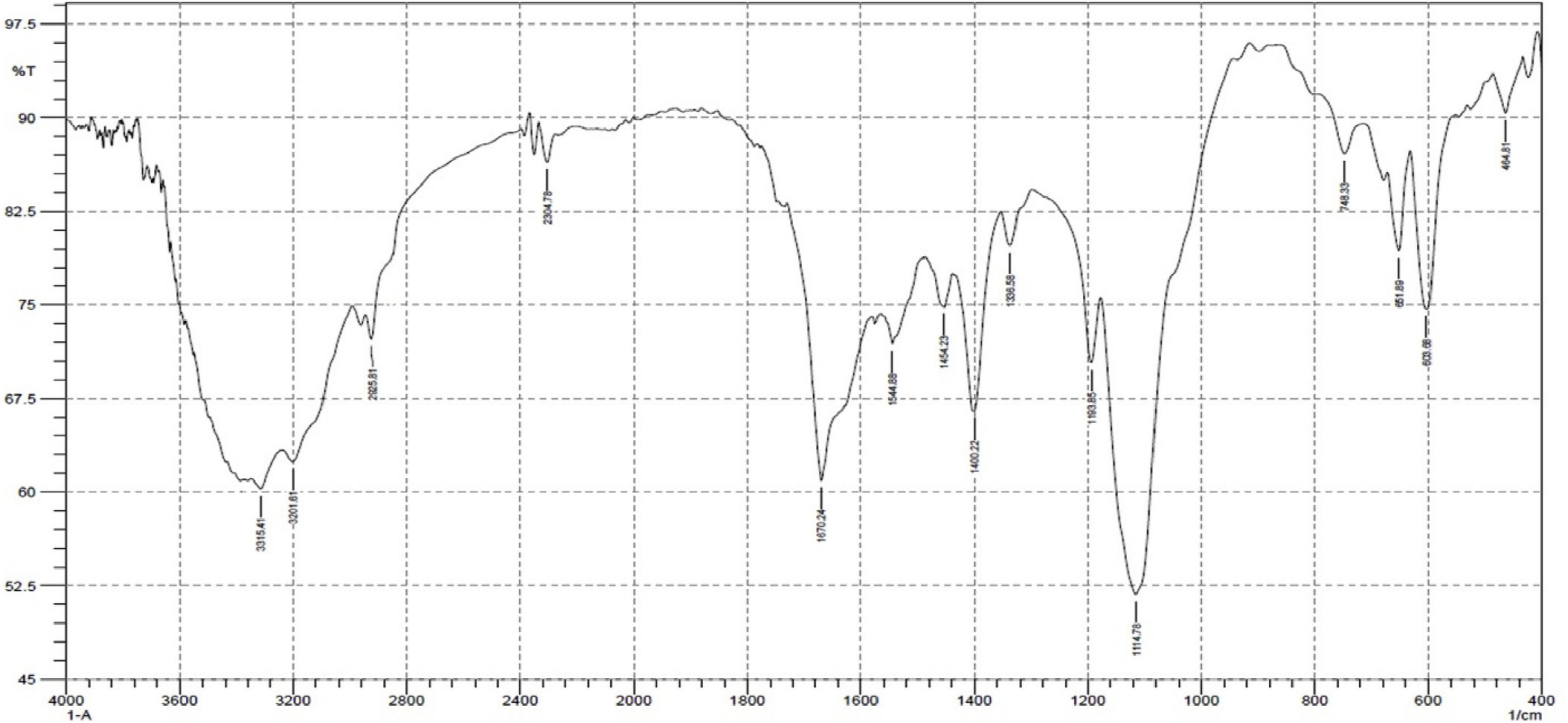
FT-IR spectrum of infected tissue (*A.hydrophila*) of *B. spirata.*

### 3.6 Amino acids analysis

The percentage composition of amino acids profile on dry matter bases of *B. spirata* presented in control and infected tissue are shown in Table 4. The total amino acids of control were found to be as 62.70% and non essential amino acids 38.07%. The essential amino acids, phenylalanine was recorded high as 19.55% on dry matter basis in control tissue and the non essential amino acids, asparagine were found maximum as 10.65% on dry matter basis in infected tissue. The total non essential amino acids of control tissue were registered to be as 63.09% and infected tissues were registered to be as 33.39%. Among the essential amino acids of infected tissue, lysine was observed high as 10.04% and among the non essential amino acids of control tissue, serine was found maximum as 11.30%.

**Table 4:** Amino acids profile of control and infected with *A. hydrophila* in *B. spirata*.

S. No.	Amino acids (%)	Control	Infected with *(A. hydrophila*)
**Essential Amino acids (EA)**			
1	Histidine	12.34 ± 0.89^a^	2.61 ± 0.40^ab^
2	Isoleucine	6.39 ± 0.47^a^	1.97 ± 0.28^b^
3	Leucine	4.39 ± 0.51^a^	4.52 ± 0.41^b^
4	Lysine	5.47 ± 0.47^a^	10.04 ± 0.31^ba^
5	Methionine	3.76 ± 0.58^a^	2.93 ± 0.09^ba^
6	Phenylanine	19.55 ± 0.67^a^	2.61 ± 0.17^b^
7	Threonine	4.12 ± 0.78^a^	4.45 ± 0.45^ab^
8	Tryptophan	2.35 ± 0.36^a^	2.20 ± 0.24^b^
9	Valine	4.42 ± 0.33^a^	2.06 ± 0.38^b^
**Non-Essential Amino acids (NEA)**			
10	Glycine	1.48 ± 0.31^ab^	7.64 ± 0.29^b^
11	Serine	2.69 ± 0.30^a^	11.03 ± 0.27^b^
12	Glutamic acids	0.47 ± 0.10^a^	5.53 ± 0.40^ab^
13	Cysteine	3.51 ± 0.32^a^	8.46 ± 0.42^b^
14	Alanine	2.39 ± 0.38^a^	7.33 ± 0.22^b^
15	Proline	5.82 ± 0.29^a^	10.74 ± 0.32^b^
16	Aspartic acid	3.57 ± 0.41^a^	5.29 ± 0.29^ab^
17	Tyrasine	3.39 ± 0.21^a^	1.35 ± 0.11
18	Asparagine	9.65 ± 0.39	3.20 ± 0.25^ba^
19	Arginine	2.04 ± 0.50^a^	2.18 ± 0.49^ba^

^abc^ (Mean ± SD) the same letter in the same row is not significantly different at P < 0.05.

### 3.7 Fatty acids analysis

The total estimation of fatty acids profile of control and infected tissues of *B. spirata* are shown in Table 5. In control tissue 19 different fatty acids were noted and they are 8 saturated fatty acids (10.40 mg/g), 7 unsaturated fatty acids (13.87 mg/g) and 4 poly unsaturated fatty acids (14.5 mg/g). Among the PUFA linoleic acids were major acids. The infected tissues availability of SFA, UFAs and PUFA contents were 8.67, 21.38 and 6.41 mg/g.

**Table 5:** Fatty acid profile of control and infected with *A. hydrophila* of *B. spirata*.

S.No	Fatty acids (mg/g)	Carbon atom	Control	Infected with (*A. hydrophila*)
**Saturated Fatty acids (SFAs)**				
1	Lauric acid	C12	0.44 ± 0.25^ab^	0.09 ± 0.04^b^
2	Myristic acid	C14	1.45 ± 0.37^a^	0.72 ± 0.14^b^
3	Pentadecylic acid	C15	0.17 ± 0.16^a^	0.37 ± 0.22^b^
4	Palmitic acid	C16	1.32 ± 0.21^a^	0.89 ± 0.15^b^
5	Margaricacid	C17	1.37 ± 0.27^ab^	0.69 ± 0.31^b^
6	Stearic acid	C18	2.85 ± 0.38^a^	2.80 ± 0.34^b^
7	Heneicosylic acid	C21	0.37 ± 0.13^ab^	0.59 ± 0.06^b^
8	Tricosylic acid	C23	2.43 ± 0.41^a^	2.52 ± 0.47^ba^
**Unsaturated Fatty acids (UFAs)**				
9	Vaccenic acid	C18:1	1.17 ± 0.27^a^	0.92 ± 0.32^b^
10	Linoleic acid	C18:2	2.18 ± 0.19^a^	2.32 ± 0.81^ba^
11	Paullinic acid	C20:1	2.37 ± 0.51^a^	2.22 ± 0.25^b^
12	Eicosadienoic acid	C20:2	0.41 ± 0.57^a^	0.65 ± 0.14^b^
13	Arachidonic acid	C20:4	1.18 ± 0.14^a^	3.85 ± 0.52^b^
14	Elcosapentaenoic acid	C20:5	1.43 ± 0.39^a^	2.84 ± 0.38^ba^
15	Erucic acid	C22:1	5.13 ± 0.27^a^	8.58 ± 0.32^b^
**Poly Unsaturated Fatty acids (PUFAs)**				
16	Linoleic acid	C18:2 (n-6)	11.32 ± 0.19^a^	2.32 ± 0.81^b^
17	Gamma- linolenic acid	C18:3 (n-6)	0.35 ± 0.17^ab^	0.45 ± 0.25^b^
18	Docosadienoic acid	C22:2	0.42 ± 0.27^a^	0.56 ± 0.63^ba^
19	Docosahexaenoic acid	C22:6	2.41 ± 0.38^a^	4.11 ± 0.04^b^

^abc^ (Mean ± SD) the same letter in the same row is not significantly different at P < 0.05.

### 3.8 Minerals content analysis

The quantities of mineral present in the control and infected tissue of *B. spirata* are shown in Table 6. In control tissue, totally 5 macro minerals and 11 micro minerals were detected. Among the macro minerals calcium (13903.33 ppm), sodium (1202.67 ppm) and phosphorous (379.33 ppm) were observed at higher and lower level levels. The micro minerals such as aluminium (74331.67 ppm), zinc (65285 ppm), and boron (11.63 ppm) were recorded at higher and lower level in control tissue. In infected tissue, totally 6 macro minerals and 12 micro minerals were detected. Among the macro minerals barium (1.93 ppm) and gallium (39.33 ppm) were presented in infected tissue alone. In control tissues, they were not present.

**Table 6:** Composition of minerals of control and infected with *A. hydrophila* of *B.spirata*.

S.No	Minerals	Control	Infected (*A. hydrophila*)
1	**Macro Minerals**	13903.33 ± 3.05^ab^	4730.66 ± 3.03^b^
	Calcium (ppm)		
2	Sodium (ppm)	12902.67 ± 2.81^a^	10060.33 ± 2.51^b^
3	Magnesium (ppm)	6202.66 ± 2.51^a^	3490.00 ± 2.00^ba^
4	Potassium (ppm)	8946.00 ± 3.00^a^	7740.00 ± 2.52^b^
5	Phosphorus (ppm)	379.33 ± 2.51^a^	201.00 ± 2.00^b^
6	Barium (ppm)	0	1.93.00 ± 0.40
7	**Micro Minerals**	201.00 ± 2.51^a^	51.00 ± 2.30^b^
	Iron (ppm)		
8	Zinc (ppm)	6.28 ± 2.30^ab^	2051 ± 1.52^b^
9	Cobalt (ppm)	2.15 ± 1.52^a^	25.33 ± 1.15^ba^
10	Chromium (ppm)	252.53^a^	650 ± 1.52^b^
11	Copper (ppm)	5692.67 ± 2.08^a^	1500.33 ± 1.52^b^
12	Manganese (ppm)	7831.66 ± 1.15^ab^	1532.33 ± 1.22^b^
13.	Gallium (ppm)	0	39.33 ± 1.51
14	Lithium (ppm)	750.33 ± 1.15^a^	250.66 ± 1.52^ab^
15	Nickel (ppm)	300.00 ± 1.73^a^	78.66 ± 1.52^ab^
16	Selenium (ppm)	2025.66 ± 2.08^a^	1562.00 ± 1.78
17	Aluminum (ppm)	74331.67 ± 1.52^a^	15841.64 ± 0.58^b^
18	Boron (ppm)	11.63 ± 1.25^a^	5.30 ± 0.57^ab^

^abc^ (Mean ± SD) the same letter in the same row is not significantly different at P < 0.05.

## 4 Discussion

The aquaculture of molluscs seems to be seriously affected worldwide by bacterial pathogens and predators’ that cause high losses in hatcheries as well as in natural beds. The main responsible for the mortality outbreaks is a number of *Vibrio* sp and *Aeromonas* species that are considered important pathogens in aquaculture [13]. Temperature and salinity are considered to be the most important physical factors influencing marine organisms and the biological effects of these factors are complex and wide ranging [14]. Similarly, in the present study observed average water quality parameters, temperature, 26.26 to 27.26 °C, salinity 7.36–7.73 ppt, the pH 32.28 to 33.36 and the dissolved oxygen 5.21–5.70 mg/L were recorded. The higher body weight gain and shell length increments were observed in *B. areolata* held in recalculating seawater system at water exchange of 15 day intervals [15]. The gut content of *B. spirata* were analyzed in the wild and cultures animals and found that heterotrophic bacteria count was more in the case of wild compared to the cultures ones (105 × 10^2^ CFU/g and 27 × 10^2^ CFU/g). *Vibrios* sp and *Micrococcus* sp were present in wild one at level of 7 × 10^5^ CFU/g and 3 × 10^2^ CFU/g respectively [16]. Similarly, in the present study with the bacterial challenges, the maximum mortality rate was observed in T_7_ (*Aeromonas hydrophila*) while, the lowest rate was observed in T_2_ and T_4_ of *B. subtilis* (30%) and *S. aureus* (30%). The antimicrobial activity of tissue extract of *B. spirata*, the maximum (12 mm) was observed against *Pseudomonas aeruginosa* and minimum (2 mm) against *Staphylococcus aureus,* molecular weight of protein profile range of this species from 2 KDa to 10 KDa with presence of bioactive compounds [17]. Similarly, the present investigation, muscle extraction of wild, control and infected tissue of *B. spirata* showed, the maximum protein was observed in wild and minimum was observed in infected tissue and were range from 14.3 KDa to 97 KDa.

DNA molecules with in cells will be chopped up into various fragments with different length, thus leading to DNA fragments with different size of base pairs and their integral times. Similarly, 100 μL of hydrogen peroxide infected with oyster (*Crassostrea gigas)* showed increase the DNA damage compared to uninfected group of bivalves [18]. The present study showed that wild and control tissue of *B. spirata* had thin bands and no DNA fragmentation. The infected group of snail tissue had observed DNA fragmentation. These results showed the wild and control tissue might provide the evidence for denaturing DNA and degrading the bacterial colony or control the bacterial growth.

Molluscs especially gastropods are widely consumed in many parts of the world by humans because they are having high protein content, low saturated fat, high trace minerals and also contain omega 3 fatty acids known to support good health. The mineral components such as sodium, potassium, magnesium, calcium, iron, phosphorous and iodine are very essential nutrient for human [19]. The *B. spirata* meat is a most important food with high quality protein and well balanced diet for human consumption, nutritional contents of this species such as protein (53.86%), carbohydrate (16.85%) lipid (9.30%) and 10 essential and non essential amino acids were recorded. The molecular weight ranged from 2 KDa to 110 KDa and FTIR spectrum showed the presence of bioactive compounds to fight against some dread full microbes [4,20].

The biochemical composition of gastropods viz., *Babylonia zeylanica, Murex virgineus, Babylonia spirata,* and *Trochus radiatus* from Kanyakumari coast. The maximum protein content was observed in *B. spirata* (39.8%) and *B. zeylancia* (39.8%) [21]. The present study showed the essential and non essential amino acids was recorded both the control and infected tissues. The total essential amino acids of control tissue were found to be (62.79%) and the total non essential amino acids of control (38.07%). Among the total amino acids, phenylanine (19.55%), asparagines (10.68%) were presented in maximum level in control tissue. The total essential amino acids of infected tissue were found to be (33.39%) and non essential amino acids (63.09%). Among the total amino acids, lysine (10.04%), serine (11.30%) and proline (10.74%) were presented in maximum level in infected tissue. These studies clearly demonstrate that *B. spirata* can be well used as the potential source of amino acid by all section of people to do way with malnutrition.

*Thais bufo* (Lamarck) of proximate composition and amino acids profile showed protein, carbohydrate and lipid were observed to be 22.34%, 19.34%, and 4.56% respectively. Almost 7 fatty acids were identified. Among them, 3 saturated fatty acids, 1 mono saturated fatty acids and 2 poly unsaturated fatty acids. In amino acids, totally 24 amino acids were identified. Among them 9 were essential and 11 were non essential amino acids [22]. The present studies have indicated that the gastropod was an imperative protein source and in future they might be used to alleviate protein scarcity problems in the developing countries.

The fatty acid profiles of gastropods are usually dominated in PUFA and the present study also shown the same where, maximum observed in *B. spirata.* Studies have found support that linolenic acid is related to lower risk of cardiovascular disease. Dietary α –linolenic acid has been assessed for its role in cardiovascular health [23]. The marine molluscs are rich source of PUFA [24]. In the present study, 19 different fatty acids were found in control tissue of *B. spirata* and they are 8 saturated fatty acids (SFA), 7 unsaturated fatty acids (USFAs) and 4 poly unsaturated fatty acids (PUFA). Among the PUFA, linolenic acids were major acids in the fatty acids group. The availability of SFA, USFA and PUFA content was 10.40, 13.87 and 14.5 mg/g respectively. In infected tissue of *B. spirata* availability of SFA, USFA and PUFA content were 8.67, 21.38 and 6.41 mg/g respectively. The fatty acid profile of *B. spirata* tissue, the saturated fatty acids were dominant fatty acids (35.28%), Mono Saturated Fatty Acids (26.57%) and PUFA (11.72%) [20]. In the present study, 19 fatty acids were recorded in the tissues of *B. spirata*. They are 8 saturated fatty acids, 7 unsaturated fatty acids and 4 poly unsaturated fatty acids.

The mollusc's shells and tissues are good indicator of metal pollution as they are sessile and sedentary and they reflect the heavy metals concentration of that particular area [25]. The mineral deficiencies can cause biochemical structure and functional pathologies which depends on several factors, including the duration and degree of minerals deprivation. During the present study totally, 5 macro and 11 micro minerals were detected in control tissue of *B. spirata*. Among the macro minerals, calcium (13903.33 ppm) and phosphorous (379.33 ppm) were observed at higher and lower level, whereas other macro minerals such as magnesium and calcium were negligible level. In infected tissue, totally 6 macro minerals and 12 micro minerals were detected. Among the macro minerals, sodium (10060.33 ppm) and barium (1.93 ppm) were observed at maximum and minimum level and the micro minerals such as copper (26.991.33 ppm) and boron (5.30 ppm) were observed in higher and lower level, whereas the barium and gallium were not present in control tissue. These are important pollutants for many aquatic organisms, the barium and gallium were present in infected snails because the water quality of this group of snails may enriched in mineral organic substance by their physiological factors. It is clearly toxic to many animal species [26].

Fourier transform infrared (FTIR) spectroscopy is a non-disturbing technique which showed quantitative bioactive profile about biological samples [27]. The present study FTIR spectrum of the control showed, 19 major peaks lying between 3315.41 cm^−1^ and 603.68 cm^−1^. whereas the spectrum of infected tissue showed the 15 major peaks lying between 3315.41 cm^−1^ and 601.68 cm^−1^. FTIR spectrum analysis showed the presence of bioactive compounds single at different from control and infected tissue. This large shift might be simply a variation in the strength of protein and amide hydrogen banding due to change in the plasma chemistries.

## 5 Conclusion

The present study showed that the low nutritional values of *B. spirata* might be due to low quality of contaminated tissue. The aquatic pathogen of *A. hydrophila* highly infects the experimental gastropods so that, it's consider as the vulnerable pathogen. It is clear that the control (uninfected) tissue of gastropod contains rich nutritive value and can be used for alternative source as regular seafood. This supplies nutrients for growing children and people suffering from malnutrition. Based on this result, the presence of rich protein content, amino acids (phenylalanine), fatty acids (linolenic acids), minerals (aluminum and copper) and good protein profile in this species add more value of economic importance to the same.

## Declaration of competing interest

There is no conflict of interest.
